# A cloud-free MODIS snow cover dataset for the contiguous United States from 2000 to 2017

**DOI:** 10.1038/sdata.2018.300

**Published:** 2019-01-15

**Authors:** Hoang Tran, Phu Nguyen, Mohammed Ombadi, Kuo-lin Hsu, Soroosh Sorooshian, Xia Qing

**Affiliations:** 1 Center for Hydrometeorology and Remote Sensing, Department of Civil and Environmental Engineering, University of California, Irvine, Irvine, California, USA; 2 Nong Lam University, Ho Chi Minh City, Vietnam

**Keywords:** Hydrology, Hydrology

## Abstract

This article presents a cloud-free snow cover dataset with a daily temporal resolution and 0.05° spatial resolution from March 2000 to February 2017 over the contiguous United States (CONUS). The dataset was developed by completely removing clouds from the original NASA’s Moderate Resolution Imaging Spectroradiometer (MODIS) Snow Cover Area product (MOD10C1) through a series of spatiotemporal filters followed by the Variational Interpolation (VI) algorithm; the filters and VI algorithm were evaluated using bootstrapping test. The dataset was validated over the period with the Landsat 7 ETM+ snow cover maps in the Seattle, Minneapolis, Rocky Mountains, and Sierra Nevada regions. The resulting cloud-free snow cover captured accurately dynamic changes of snow throughout the period in terms of Probability of Detection (POD) and False Alarm Ratio (FAR) with average values of 0.955 and 0.179 for POD and FAR, respectively. The dataset provides continuous inputs of snow cover area for hydrologic studies for almost two decades. The VI algorithm can be applied in other regions given that a proper validation can be performed.

## Background & Summary

The Moderate Resolution Imaging Spectroradiometer (MODIS) snow cover area (SCA) product^1^ serves as a reliable source of snow measurements for hydrologic studies as well as for data assimilation in climate models. Researchers used and evaluated the product in various regions including the Columbia and Missouri river basins^2^, Austria^3^, Xinjiang, China^4^, and in Sierra Nevada^5,6^. The datasets were also incorporated into a land surface model^7^ and a hydrology model^8^ for data assimilation.

Nevertheless, cloud obscuration limits the product's usage. Clouds block satellites from capturing the ground state of the earth surface (i.e. snow/land), especially during snow accumulation periods when clouds gather and block the snow cover extent information from the ground. Major concerns about MODIS snow maps polluted by clouds and snow/cloud discrimination have been repeatedly mentioned in its assessment studies^9,10^ as well as in the MODIS product user guide^11^. Hence, researches focus on the removal of clouds from snow images have been conducted for years and have been classified into two main categories: model-driven methods and data-driven methods. To estimate snow extent beneath clouds, the model-driven methods rely on the relationships between snow and other factors such as: energy and mass exchanges^12,13^, temperature^14^, and grain size^15^.

On the other hand, data-driven methods take into account the persistent characteristics of snow to remove clouds. For example, Dozier *et al.*^16^ considered snow data as a sparse space-time cube that could be filled by temporal cubic spline interpolation. Their results demonstrated that the interpolated and smoothed product has more consistent snow-covered area in the Tuolumne and Merced River basins throughout the water year 2005 than the raw, cloud cover filtered data. However, Dozier *et al.* approach is limited to 1-D interpolation primarily because of frequent zenith angles oscillation and slow computation. Later on, Gafurov and Bardossy^17^ introduced a series of six spatiotemporal filters to mitigate cloud cover from MODIS images in the Kokcha River basin in Afghanistan, detailed information of this study will be presented in subsequent sections. Parajka *et al.*^18^ proposed a regional snow-line method (SNOWL) utilizing elevation information for de-clouding; the method robustly recovered snow cover maps from cloud over Austria^18^. In another study, Hall *et al.*^19^ suggested a cloud-gap-filled (CGF) method to produce a snow cover map with cloud-persistence count (CPC) for each grid where lower CPC snow grids are more likely to have snow. While approaches from Parajka *et al.*^18^ and Hall *et al.*^19^ are simple and proven to be suitable for use in hydrological and global models, persistent cloudy conditions during snow accumulation periods may reduce their reliability significantly. More recently, Dong *et al.*^20^ employed information from snow stations to estimate ground states of cloud-cover areas from MODIS in south-western Germany. Although this approach is effective in areas with dense snow networks, it requires manually determining thresholds for each stationʼs predicting capability of nearby snow cover based on station location and elevation.

To completely remove clouds and delineate dynamic snow boundaries, Xia *et al.*^21^ implemented the Variational Interpolation (VI) method^22^ for interpolating the three-dimensional space-time cube of snow cover proposed by Dozier *et al.*^16^. Evaluation results in the Sierra Nevada mountain range demonstrated that the method was robust and accurate since during the accumulation period (25–27) March 2007, the “Cleared” images obtained from VI had an average omission error and commission error of about 22.5 and 2.1% respectively since the initial omission errors from the original Terra and Aqua images were 14.3 and 20.2% respectively. Meanwhile during the melting period (14–16) March 2009, original satellite observations were not obscured by clouds as severely as accumulated ones, hence the errors of omission and commission between ʽClearedʼ and MODIS images were similar (5.7 to 5% of omission errors and 0% for commission errors). However, the main drawback of the original VI method is the system instability which limits its implementation on a larger scale. In this study, an improved VI version is introduced by integrating MINimum RESidual (MINRES) iterations^23^ to prevent the system from breaking up when applied to much broader scales.

The procedure of developing the cloud-free snow cover dataset consists of two parts. First, estimation of the ground states (i.e. snow/land) of cloud-hindered grids via five filters adopted from Gafurov *et al.*^17^. The resulting images are still obstructed by clouds but provide more details about snow boundaries which is beneficial to the second step. In the second stage, the improved VI method is implemented to reconstruct a three-dimensional time-varying snow cover boundary. Subsequently, this 3-d surface can be used to obtain cloud-free snow cover images.

## Methods

The spatial domain of the dataset developed in this study is the contiguous United States (CONUS) which covers about 8,080,464.3 km^2^, ranges between 24^o^30N and 49^o^25N in latitude and from 66^o^57W to 124^o^46W in longitude. During winter seasons, from November to the end of February, the snow cover extent for the whole CONUS varies from one million km^2^ to four million km^2^ which plays a crucial role in energy and hydrological cycles^24,25^.

The main inputs in this study are products from MODIS/Terra Snow Cover Daily (MOD10C1) and MODIS/Aqua Snow Cover Daily (MYD10C1) version 6 (Data Citation 1) released in July 2016 by the National Snow & Ice Data Center (NSIDC). These daily Climate Modeling Grid (CMG) products are in a sequence of MODIS snow product suite^11^, beginning with the 500 m resolution swath product (MOD10_L2).

As reported in Riggs and Hall^11^, the swath level snow mapping algorithm is based on the Normalized Difference Snow Index (NDSI)^26,27^. NDSI is calculated for Terra/MODIS using band 4 and band 6, for Aqua/MODIS using band 4 and band 7.

(1)NDSI=Band4 − Band6Band4 + band6

The global criteria for snow is NDSI greater than 0.4 and near-infrared reflectance (band 2) greater than 0.11 and band 4 reflectance greater than 0.10. To increase snow detection sensitivity in forested landscapes, the MOD10_L2 product combines the Normalized Difference Snow Index (NDSI) range from 0.1 to 0.4 with the Normalized Difference Vegetation Index (NDVI)^11^. After a pixel is classified as snow, “it is subjected to a series of screens to alleviate snow commission errors and flag uncertain snow detections”^11^. More details about the screens can be found in the product user guide^11^. Here, we only summarized the screens main thresholds: (1) Version 6 combines surface temperature and height screen, if snow pixels are in low elevation (<1300 m) and warm surfaces (>283 K), they are reversed to no-snow. This new surface temperature screen solves the problem of detecting snow in mountain ranges during spring and summer brought up by Rittger *et al.*^5,10^; (2) If snow pixels have low reflectance (Very High Visible (VIS) of band 2 is ≤0.10 or band 4 is ≤0.11) or low illumination (solar zenith angles > 70°), they will be set as no-snow or night pixels; (3) Low NDSI or unusually high Short-Wave Infrared (SWIR) reflectance snow pixels will be converted back to no-snow pixels.

The next product in the sequence, MOD10A1, only selects one 'best' observation from all the MOD10_L2 swaths over a location using strict criteria including solar elevation, distance from nadir, and observation cover. Selecting an observation closest to nadir with maximum coverage of the cell^9^ could solve problems from the off nadir viewing from the MODIS reflectance product (MOD09) mentioned by Dozier *et al.*^16^.

The main input of the study, MOD10C1/MYD10C1, “maps 500 m MOD10A1 observations into 0.05^o^ CMG cells. Outputs for a grid cell are determined by the percentage of counts of observations, snow or cloud, mapped in the cell”^11^. The daily MODIS snow product suite has “an overall accuracy of about 93%, lower accuracy is found in forested areas”^9^. More recent studies from Liu *et al.*^26^ and Rittger *et al.*^5^ also highlighted scenarios in which dense canopy limits the product ability to detect snow cover.

It should be noted that the launch dates of the two satellites, Terra and Aqua, are different with December 18, 1999 and May 4, 2002 for Terra and Aqua respectively. In this study, the term MODIS-SCA product will be used to represent both products, MOD10C1 and MYD10C1, from Terra and Aqua whenever the text refers to 2002 or later. When referencing prior to 2002, the term only represents products from Terra satellite.

Overall, the process of creating the cloud-free product from the MOD10C1/MYD10C1 product starts with reclassifying MODIS into one of three categories, namely snow, land (no-snow), and cloud based on the threshold of 50% fractional: if a grid has a percentage of snow greater than 50%, it is set as snow. If the sum of the snow and cloud fractions in one location is smaller than 50%, a grid is marked as land (no-snow). If neither snow nor no-snow, a grid is set as cloud. Next, the reclassified MODIS images passed through two subsequent steps based on a series of filters and the VI algorithm. These two steps are discussed in the following subsections.

### Mitigated filters

**Figure 1:**
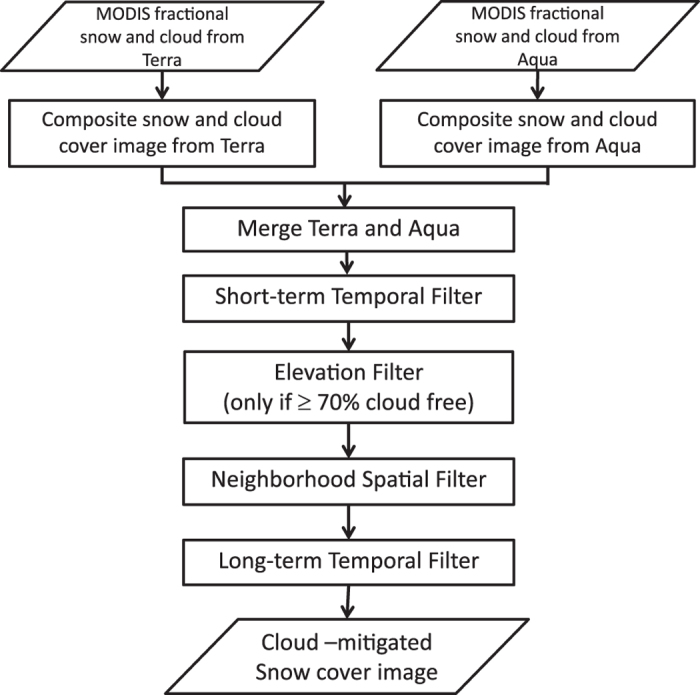
Flow chart of cloud mitigation filters. The process consists of five filters: (1) combining Terra and Aqua snow cover images in a same day, (2) short-term temporal filter, (3) elevation filter, (4) neighborhood spatial filter, and (5) long-term temporal filter.

The filters are used as a first step to retrieve cloud-free snow cover images. This method has been adopted from Gafurov and Bardossy^17^ and it consists of five filters to mitigate cloud obstruction: (1) combining Terra and Aqua snow cover images in a same day, (2) short-term temporal filter, (3) elevation filter, (4) neighborhood spatial filter, and (5) long-term temporal filter. Fig. 1 illustrates the flow of cloud polluted images through the filters and the functions applied by the filters. The importance of using the filters is that they provide the necessary information about snow boundaries in order for the VI algorithm to be applied.

The first filter implies an assumption that no snowmelt or snowfall occurred within two observations of MODIS in one day. Thus, as long as one satellite views a pixel as snow (or land), this ground status will be assigned to the pixel in the combined image. The formula is given as follows in equation (2):

(2)S(x,y,t)=max(S(x,y,t)A,S(x,y,t)T)

Where x and y are spatial (i.e. longitude and latitude) coordinates of pixel S; t is the day index of pixel S. S^A^ and S^T^ represent pixels from Aqua and Terra respectively. The second filter assigns the cloud grids as snow (or land) if the cloud covered pixel showed both snow (or land) in the preceding and succeeding days. This step is formulated as equation (3):

(3)S(x,y,t)=1if(S(x,y,t−1)=1andS(x,y,t+1)=1)

In the third filter, a maximum and a minimum elevation lines defined as the highest and lowest elevation of snow grids in the image are determined. To ensure the snow lines are correctly determined, the condition of this filter is that at least 70% of the image is cloud free^17^. Otherwise this filter will be skipped. The filter assigns grids with a lower elevation than the minimum elevation line $H_{min}^{S}(t)$ as land. Likewise, grids with a higher elevation than the maximum elevation line $H_{max}^{S}(t)$ are assigned as snow. The formulas are given as follows in two equation (4) and (5):

(4)S(x,y,t)=0if(H(x,y)<HminS(t))

(5)S(x,y,t)=1if(H(x,y)>HmaxS(t))

where H_(x,y)_ is the elevation of a pixel (x,y) location. The last two filters apply spatial and temporal processing respectively. Firstly, the fourth filter merges two neighborhood spatial filters of Gafurov and Bardossy into one. Specifically, if three out of four direct “side-bordering” pixels of the cloudy pixel indicate snow/land, the cloudy pixel will be set as snow/land. Furthermore, when considering all eight neighboring pixels, if any pixel both has lower elevation than the centre elevation and shows snow, the center pixel will also be assigned as snow. This step is formulated as equation (6):

(6)S(x,y,t)=1if(S(x+k,y+k,t)=1andH(x+k,y+k)(k ∈(−1,1))<H(x,y))

In the last filter, a new long-term temporal filter was developed based on the fact that the annual snow-status time series can be separated into three types of periods: snow, land, and transition periods. For a snow period, the grids either show snow or cloud, therefore, cloudy pixels would be assigned as snow. On the other hand, for a land period, the grids either show land or cloud, therefore, cloudy pixels would be assigned as land. The selection of length of the period is subjective, but must be long enough to avoid phase change or long-lasting cloudy periods. For this study, we chose a 30-day window period. Interested readers should refer to (Gafurov and Bardossy^17^) for detailed information about the filters.

### Variational Interpolation algorithm

The time-varying snow cover boundaries resulted from previous filters are modeled by the VI algorithm^22^ using a three-dimensional implicit function formulated as:

(7)f(x→){>0=0 <0 insidesnowcoveratsnowboundariesoutsidesnowcover

where $\vec{x}={\left(x_{1}\;x_{2}\;t\right)}^{T}\in R^{3}$, x_1_ and x_2_ are spatial coordinates on the projection plane, and t is the time. In three spatial dimensions, implicit functions deliver relatively simple techniques to generate complicated but useful surfaces^27^. Once the snow cover implicit surface in space and time is determined, cloud-free images from selected days can then be obtained through cross sections of the surface^21^.

One thing to note is that interpolation from implicit surfaces depends heavily on the surface smoothness. In order to apply VI for snow cover, we have to make a hypothesis about the dynamic property of snow cover boundaries. Numerous theories in Physics such as the Principle of Least Action^28^, Principle of Least Forcing^29^, and the Variational Principle^30^ proved that a natural process always operates in its most efficient way. As energy cost is one of the most crucial factors of efficiency, a natural surface should hold the minimum energy cost. Hence, it can be represented as a linear combination of the radial-basis function established at selected constraint points on the surface according to the following equation^21,^^31^:

(8)f(x→)=0⇒∑i=1NwiR(x→−xi→)=0

where **w** is a set of *N* weights and $R\left(\vec{x}-\overset{\to }{x_{i}}\right)$ is a selected radial-basis function established at *N* constraints points. We decided to use the thin plate function *R*(.) = *r*^2^ log r with $r=\vec{x}-\overset{\to }{x_{i}}$ to present the radial-basis function. With constraint points collected on snow boundaries in discrete times, the weights of those points can be computed by solving the linear system to create the implicit surface.

To provide VI with necessary constraint points, the Douglas-Peucker algorithm has been used^32^. This one parameter method is simple and widely used in vector graphics simplification and cartographic generalization. Given a relative distance dimension ε (0<*ε*<1) and a starting curve of an ordered set of points, the algorithm recursively divides the curve to discard points closer than ε to line segments. The larger ε, the less points will be kept^32^. After experimenting a wide range of ε, a value of *ε*=0.2 has been chosen to preserve shapes of snow boundaries and reduce the number of points fed into the VI algorithm.

### Improving the system stability for the VI algorithm

The performance of the original VI algorithm^21^ is unstable when a massive amount of constraint points is collected. For example, if constraint points were collected for the CONUS region in January 2009 (i.e., ~130,000 points), the original linear system (3) will become singular and the whole system will break down. A proposed solution for this problem is using MINRES algorithm by Paige and Saunders^23^. MINRES is a Krylov subspace method for solving large symmetric systems. When applied to an inconsistent system (i.e., a singular symmetric linear problem), Paige *et al.*^33^ and Choi^34^ reported that MINRES maintains the system stability and provides a least-squares solution.

Paige *et al.*^33^ analyzed the convergence behavior of the MINRES method in singular systems and concluded its residual monotonically decreases toward the origin satisfying several convergence properties. Interested readers should refer to Paige *et al.*^33^.

**Figure 2:**
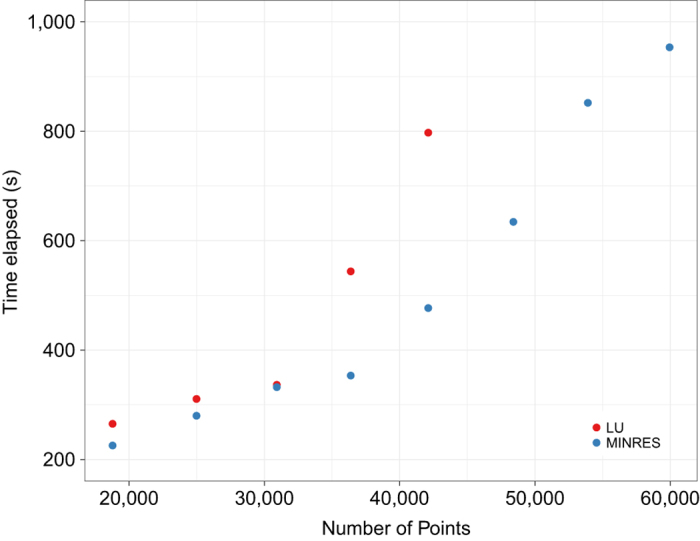
Results of computational efficiency comparison between the VI algorithm integrated with LU decomposition and the MINRES iteration. Computation time to solve a linear equation using LU decomposition (red) and MINRES iteration (blue) regarding the number of points collected into the equation.

In our implementation of the MINRES algorithm, to ensure both the system stability and accuracy, besides setting the tolerance threshold of 1e-08 (i.e. the smaller the threshold is, the more reliable the results become), we also specified the maximum number of iterations as 1000. Hence, when either of these conditions was reached, the algorithm terminated. In a small experiment, we compared the system stability of VI using both traditional LU decomposition and the MINRES method over the CONUS region in January 2009. The performance was measured by the time elapsed over the increment of the interpolation period. Both methods started by using five consecutive days as a calculation unit. However, as more points were collected, the ordinary LU decomposition resulted in a system broke down (Fig. 2). In contrast, the MINRES method demonstrated its superiority by maintaining the system stability and accelerating the computation time. It is worth noting that, in all singular cases, the MINRES terminated before reaching the maximum number of iterations. This termination indicated that the system accuracy was also guaranteed.

### Creating the cloud-free dataset

The cloud-free dataset was developed by applying the mitigating filters and the VI algorithm introduced in the previous subsections. Regarding the use of VI algorithm, wide ranges of interpolation periods were examined to obtain the optimum results in terms of accuracy; a period of 30 days has been selected. This interpolation period is used in a moving window approach such that the window is centered around the day under consideration in order to efficiently utilize information about snow boundaries. The interpolation period started from February 24, 2000; the whole dataset was created in more than one month, using the High Performance Clusters (HPC) of the University of California, Irvine.

### Code availability

The code is developed using Python 2.7 programming language; specific toolboxes used for code development include image processing toolbox (http://scikit-image.org/) and scientific computing toolbox (https://www.scipy.org/).

Due to the immense computation and the required memory size, the code was developed to be implemented in High Performance Clusters (HPC) of the University of California, Irvine. Therefore, sharing the code is of limited practical usage. However, interested readers who plan to develop cloud-free snow product using the approach described in this article can contact the corresponding author. The corresponding author will help in the adaptation of code to other computing systems.

Moreover, we are planning to build a complete package that fully utilizes the code. Any update can be found on the corresponding author's github page (https://github.com/hoangtv1899).

## Data Records

The cloud-free snow cover dataset is available to the public through an unrestricted data repository hosted by Figshare (Data Citation 2).

The characteristics of the dataset are provided in Table 1. It should be noted that there is a number of 12, 17, and 9 days missed in the years 2000, 2001, and 2002 respectively due to the unavailability in the original MODIS record.

## Technical Validation

In this study, Landsat 7 ETM+ was used as a baseline to validate the snow cover dataset spatial continuity since Landsat has high spatial resolution (30 m) and full coverage of the dataset time range (2000 to 2017). To avoid a potential saturation of ETM+ visible bands^15^, only Landsat 7 Tier 1 images have been utilized. Data from October to March of each year with a cloud threshold of less than 15% was obtained from the U.S. Geological Survey website (https://landsat.usgs.gov). Landsat 7 Tier 1 product ensures the highest available data quality for time-series processing analysis (LEDAPS^35^). Tier 1 Landsat data has RMSE no greater than 12 m and can be considered consistent and inter-calibrated across the full collection (https://landsat.usgs.gov).

In order to map snow cover from Landsat images, the SNOWMAP algorithm^26,^^36^ was used. It is based on the NDSI index which is calculated for Landsat using band 2 and band 5:

(9)NDSI=Band 2 − Band5Band2 + band5

When the NDSI is greater than or equal to 0.4 and band 4 reflectance value is greater than 11%, the pixel is classified as snow^37^. The resolution of the Landsat snow cover is then up-scaled into 0.05^o^ and reprojected into the geographic coordinate system with the spheroid of WGS84 to match with the MODIS-SCA product resolution and projection. The evaluation was conducted in four regions with different climate condition, elevation, and land cover, namely, the Seattle region, the Minneapolis region, the Rocky Mountain, and the Sierra Nevada of California. The subsequent subsection illustrates the four regions and their characteristics.

### Validation regions for snow-covered area

Landsat images from March 28, 2000 to February 15, 2017 were used for validation (See Table 2). The four selected regions for validation in this study include two high altitude regions, namely the Rocky and Sierra Nevada Mountains. The Rocky Mountains located between 40.80^o^ and 42.73^o^N in latitude, 105.51^o^ and 108.25^o^W in longitude. It has a high average altitude of 2500 m with a large area of grasslands. The region's snow regime is “predominantly continental with some pockets of intermountain characteristics”^15^. On the other hand, the Sierra Nevada Mountains (37.95^o^ to 39.89^o^N, 118.84^o^ to 121.45^o^W) is under great influence of maritime snow climates. Elevation ranges from 2m at the foothills to around 2800 m on the mountain (in the sample area) with equal portions of needle leaf forest, savannas, and grasslands.

Moreover, two additional regions were selected for validation. Firstly, a region around Minneapolis located between 43.62^o^ and 45.60^o^N in latitude, 92.09^o^ and 94.97^o^W in longitude was selected. It has an average elevation of 330 m and a land use pattern primarily consisting of cropland/natural vegetation mosaic and urban. Snow is the main form of precipitation from November through March with an annual state-wide average of 110 snow-cover days. Secondly, a region around Seattle located between 46.46^o^ and 48.42^o^N in altitude, 123.32^o^ and 123.04^o^W in longitude was selected. The region is known for its rainy climate with the Cascade Mountain range located on the east side, winters in this region are typically wet with significant snow accumulation in the mountain area. Land cover is mostly evergreen needle leaf forest and mixed forest.

From these four regions, we selected a final set of 50 tiles from over 150 Landsat tiles for best representing an overall snow climate. This selection is affected by a great variation in number of qualified Landsat tiles for each regions. In regions where snows occur frequently (i.e. Rocky Mountain, Sierra Nevada, and Minneapolis), there were small variations in Landsat tiles during each snow seasons, especially during snow melt periods when there were less clouds. On the contrary, in the Seattle region, there were many fewer Landsat tiles which were not contaminated by clouds but also contained significant snow boundaries.

### Validation results

We compared the performance of the cloud-free with Landsat using two categorical validation indices, Probability of Detection (POD) and False Alarm Ratio (FAR).

(10)POD=HitHit + Miss

(11)FAR=FalseHit + False

Over the validation scenarios, POD ranged from 0.860 to 1.000 with an average of 0.955. Modest results of POD with a mean of 0.888 came from the Seattle region since this area has a complex topography and dense forests which hindered the original MODIS-SCA product snow detection. Meanwhile, regions in high elevations or frequent snow areas showed high POD. Since the VI algorithm retrieved ground states of cloud hindered pixels, it is also important to validate the FAR of the cloud-free dataset. Across 50 validation scenes, the dataset yielded a reasonable average FAR of 0.179 with the highest FAR of 0.28 for 2 days, March 28, 2004 and March 29, 2016, in the Seattle region. The dataset modest performance in the Seattle region is justified by the rapid-varied topography and dense needle leaf forests in this region. These characteristics impose difficulties in mapping snow for this region from satellites^5,9,26,38^.

**Figure 3:**
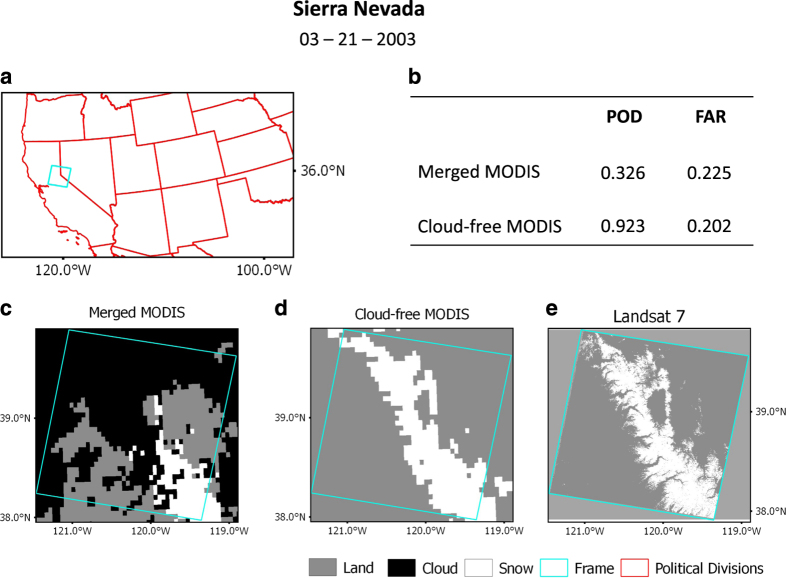
A validation example over the Sierra Nevada region on March 21, 2003. (**a**) Base map with state lines in red and a cyan rectangle to indicate the validation region. (**b**) Table showing Probability of Detection (POD) and False Alarm Ratio (FAR) results. (**c**) Merged MODIS image. (**d**) Cloud-free MODIS image. (**e**) Landsat7 image.

In Table 2, we also computed the percentage of cloud in the combined MODIS images from Terra and Aqua for each region to demonstrate the effect of the VI algorithm. In general, since we only selected Landsat images with no or low cloud percentage, the corresponding merged MODIS images are likely to contain less cloud (Table 2). However, days when cloud cover polluted the merged MODIS images heavily (e.g., January 30 and December 15, 2008 in Minneapolis or March 21, 2003 in Sierra Nevada), the VI algorithm effectively recovered snow boundaries to correlate well with the Landsat snow-cover maps. Figure 3 shows an example of snow boundary recovered from cloud cover using VI to match with the Landsat image.

### Bootstrap testing

In order to evaluate the accuracy of the mitigated filters and the VI algorithm, a cross-validation method is used. The validation process consists of three steps. Firstly, a record of synthetic cloud-covered images was developed. This was performed by selecting combined MODIS snow images over CONUS that satisfies two conditions, namely 20% or less cloud cover area and 4% or more snow cover area. Subsequently, these images were overlaid by cloud cover extracted from MODIS images with a cloud pollution rate higher than 80% to create the synthetic record. Secondly, each filter and the VI algorithm was applied sequentially to remove clouds from images in the synthetic record. Thirdly, the resulting image after each step was evaluated using the previously selected low cloud cover MODIS images. Table 3 shows the evaluation results for each filter and the VI algorithm for each day averaged across different cloud cover scenarios.

The metrics used for evaluation include POD, FAR and cloud removal ratio. The results shown in Table 3 demonstrates that each filter contributes to the cloud removal while maintaining high accuracy (i.e. POD and FAR). After complete removal of clouds, the average accuracy metrics of the images are 0.907 and 0.031 for POD and FAR respectively. Furthermore, it can be seen that the VI algorithm generally is the main factor in clouds removal with an average percentage of 0.278. Despite of this high cloud removal ratio, the VI algorithm maintains high accuracy with an average change of −0.075 and +0.014 in POD and FAR respectively.

## Usage Notes

The dataset produced in this study is useful in hydrological studies due to its adequate resolutions and validated accuracy as discussed in the previous section. In this section, we provide two simple applications of the dataset to serve as an example of the potential usages.

### CONUS Snow Cover Extent

**Figure 4:**
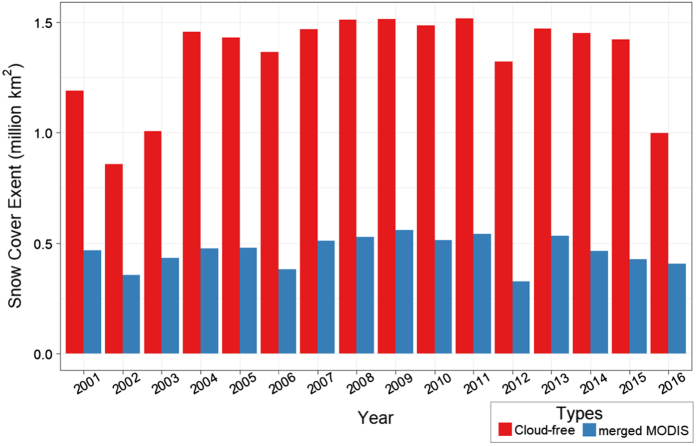
Comparison of annual average snow cover extent area over the CONUS computed from merged MODIS and the cloud-free snow cover product. The figure compares the annual average snow cover extent area over CONUS (in million km^2^ ) as computed from (**a**) the cloud-free snow cover product developed in this study (red bars) and (**b**) merged MODIS product (blue bars). The time period of the comparison is from 2001 to 2016.

The annual average snow cover extent over CONUS, measured in million square kilometers, from both the merged MODIS and cloud-free snow cover datasets was compared. The comparison was performed from 2001 to 2016. Results show that statistics of snow cover using the merged MODIS could be substantially different from the cloud-free maps. As shown in Fig. 4, the merged MODIS images (blue) show significantly less snow cover extent than the cloud-free dataset (red). The cloud-free dataset maintains an average snow cover of 1.342 million km^2^ in the period (2001–2016) compared to 0.462 million km^2^ from merged MODIS. This shows that using this dataset has major implications in quantifying the amount of snow for different hydrologic processes.

### Annual number of snow days

**Figure 5:**
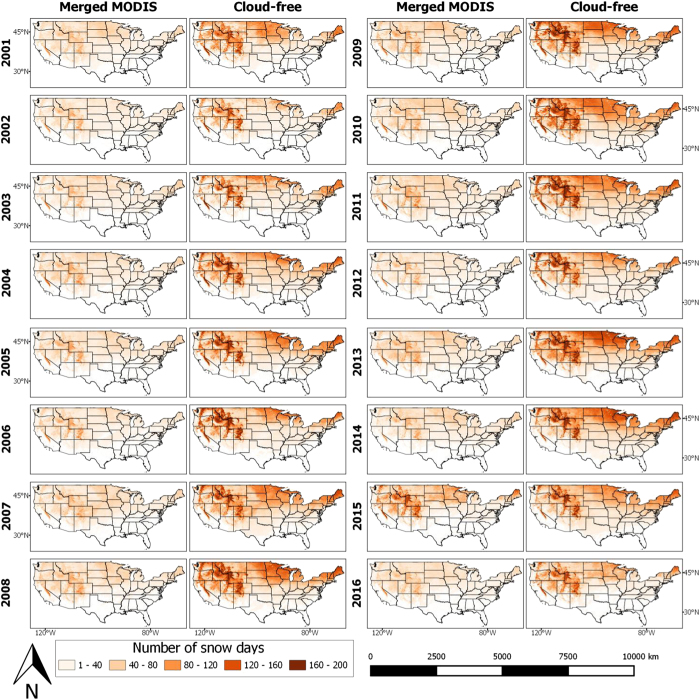
Comparison of the annual number of snow days over CONUS computed from merged MODIS and the cloud-free snow cover product. The figure compares the annual number of snow days over CONUS during the period 2001–2016 as computed from (**a**) Merged MODIS product and (**b**) the cloud-free snow cover product developed in this study.

In order to examine the differences in the two datasets beyond the yearly and monthly average of snow extent, we also studied the number of snow days over CONUS. From this perspective, we could compare the snow spatial distribution of the two products. As shown in Fig. 5, during the period (2001–2016), the merged MODIS estimates 100 days as the annual number of snow days in the mountainous area of the Western US which has an altitude range of (910–1830 m). However, the cloud-free images estimate the number of snow days in the mountain states and the Sierra Nevada area as 160 to 200 days per year.

In two drought years, 2002 and 2003, there was considerably less snow days in the western mountains and the Midwest states, such as Colorado, Wyoming, and South Dakota, as illustrated by the cloud-free maps. Moreover, the images also show shorter snow seasons from the Northeast to the Northwest of the CONUS.

The cloud-free dataset provides considerably different estimates regarding both the amount of snow as well as the number of snowy days. This has considerable implications in the results of hydrologic and climate modeling studies.

## Additional information

**How to cite this article**: Tran, H. *et al*. A cloud-free MODIS snow cover dataset for the contiguous United States from 2000 to 2017. *Sci. Data*. 6:180300 doi: 10.1038/sdata.2018.300 (2019).

**Publisher’s note**: Springer Nature remains neutral with regard to jurisdictional claims in published maps and institutional affiliations.

## Supplementary Material



## Figures and Tables

**Table 1:** Descriptive characteristics of the cloud-free snow cover dataset.

Characteristic	Variables
Data type	Binary data (0 - for ground and 1 - for snow)
Data format	NetCDF
Spatial Coverage	23.90^o^N–50.30^o^N; 130.75^o^W–63.95^o^W
Spatial Resolution	0.05^o^ × 0.05^o^
Temporal Coverage	March 10, 2000 to February 28, 2017
Temporal Resolution	1 day

**Table 2:** Landsat validation for the performance of cloud-free dataset.

Region	Landsat no. and path/row	Date	POD	FAR	Initial cloud ratio in MODIS
Seattle	L7: 46/27	03/28/2004	0.886	0.280	0.035
		10/04/2009	0.896	0.130	0.012
		03/24/2014	0.886	0.236	0.133
		03/29/2016	0.882	0.280	0.027
Minneapolis	L7: 27/29	01/10/2001	1.000	0.130	0.063
		02/27/2001	0.999	0.019	0.285
		03/15/2001	0.988	0.005	0.762
		02/17/2003	0.864	0.063	0.288
		12/23/2005	0.996	0.210	0.223
		01/27/2007	0.994	0.270	0.777
		01/14/2008	1.000	0.195	0.103
		01/30/2008	0.982	0.220	0.856
		12/15/2008	0.999	0.245	0.858
		02/01/2009	1.000	0.186	0.001
		01/06/2011	0.993	0.268	0.110
		03/27/2011	0.959	0.208	0.110
		03/06/2015	0.916	0.246	0.140
		12/03/2015	1.000	0.269	0.021
Rocky	L7: 35/31	01/02/2001	0.994	0.111	0.021
		03/07/2001	0.948	0.168	0.179
		01/13/2005	1.000	0.178	0.113
		12/15/2005	0.995	0.175	0.440
		12/18/2006	1.000	0.228	0.250
		01/22/2008	0.998	0.086	0.046
		03/10/2008	0.981	0.192	0.164
		12/23/2008	0.999	0.215	0.358
		03/29/2009	0.950	0.246	0.389
		11/24/2009	0.993	0.215	0.083
		12/10/2009	0.991	0.134	0.052
		02/15/2011	0.964	0.149	0.115
		02/18/2012	0.998	0.095	0.104
		01/03/2013	1.000	0.134	0.063
		12/05/2013	0.995	0.092	0.072
		01/06/2014	1.000	0.222	0.265
		03/30/2015	0.903	0.149	0.011
		12/27/2015	1.000	0.120	0.024
		02/15/2017	0.860	0.240	0.169
Sierra Nevada	L7: 43/33	03/28/2000	0.924	0.127	0.183
		02/27/2001	0.904	0.144	0.033
		03/05/2003	0.909	0.204	0.033
		03/21/2003	0.923	0.202	0.652
		03/07/2004	0.935	0.135	0.023
		03/10/2005	0.940	0.162	0.023
		02/15/2008	0.909	0.250	0.039
		03/02/2008	0.902	0.151	0.100
		03/18/2008	0.933	0.181	0.221
		03/05/2009	0.985	0.155	0.176
		03/16/2013	0.928	0.230	0.040
		03/19/2014	0.878	0.224	0.256
		03/24/2016	0.889	0.164	0.464
			0.955	0.179	0.199

**Table 3:** Cross validation for each filter.

Days	Filters	Percentage of Cloud Elimination	POD	FAR
Jan 24, 2006	Short-term temporal filter	0.071	0.985	0.016
	Elevation filter	0.021	0.983	0.017
	Spatial filter	0.105	0.981	0.017
	Long-term temporal filter	0.465	0.970	0.025
	VI algorithm	0.339	0.826	0.028
Jan 26, 2007	Short-term temporal filter	0.101	0.983	0.012
	Elevation filter	0.021	0.981	0.013
	Spatial filter	0.104	0.980	0.013
	Long-term temporal filter	0.397	0.981	0.013
	VI algorithm	0.376	0.953	0.017
Jan 31, 2009	Short-term temporal filter	0.135	0.984	0.013
	Elevation filter	0.025	0.981	0.014
	Spatial filter	0.080	0.980	0.014
	Long-term temporal filter	0.447	0.979	0.014
	VI algorithm	0.313	0.944	0.016
Mar 18, 2009	Short-term temporal filter	0.317	0.964	0.031
	Elevation filter	0.049	0.956	0.031
	Spatial filter	0.017	0.954	0.031
	Long-term temporal filter	0.530	0.949	0.031
	VI algorithm	0.087	0.867	0.035
Jan 18, 2013	Short-term temporal filter	0.234	0.988	0.010
	Elevation filter	0.037	0.988	0.010
	Spatial filter	0.050	0.986	0.011
	Long-term temporal filter	0.422	0.978	0.016
	VI algorithm	0.256	0.932	0.025
Mar 13, 2014	Short-term temporal filter	0.066	0.986	0.021
	Elevation filter	0.012	0.982	0.022
	Spatial filter	0.116	0.980	0.022
	Long-term temporal filter	0.453	0.965	0.049
	VI algorithm	0.353	0.917	0.051
Mar 6, 2015	Short-term temporal filter	0.146	0.986	0.012
	Elevation filter	0.035	0.981	0.013
	Spatial filter	0.090	0.979	0.014
	Long-term temporal filter	0.418	0.976	0.012
	VI algorithm	0.310	0.931	0.018
Mar 7, 2015	Short-term temporal filter	0.451	0.980	0.030
	Elevation filter	0.065	0.975	0.031
	Spatial filter	0.000	0.973	0.031
	Long-term temporal filter	0.377	0.971	0.028
	VI algorithm	0.107	0.933	0.029
Nov 23, 2015	Short-term temporal filter	0.107	0.985	0.013
	Elevation filter	0.020	0.980	0.014
	Spatial filter	0.094	0.979	0.014
	Long-term temporal filter	0.416	0.973	0.056
	VI algorithm	0.364	0.863	0.063
